# Tumorigenic Aspects of MSC Senescence—Implication in Cancer Development and Therapy

**DOI:** 10.3390/jpm11111133

**Published:** 2021-11-02

**Authors:** Slavko Mojsilović, Aleksandra Jauković, Tamara Kukolj, Hristina Obradović, Ivana Okić Đorđević, Anđelija Petrović, Diana Bugarski

**Affiliations:** Group for Hematology and Stem Cells, Institute for Medical Research, National Institute of Republic of Serbia, University of Belgrade, 11129 Belgrade, Serbia; aleksandra@imi.bg.ac.rs (A.J.); tamara.kukolj@imi.bg.ac.rs (T.K.); hristina.obradovic@imi.bg.ac.rs (H.O.); ivana.okic@imi.bg.ac.rs (I.O.Đ.); andjelija.petrovic@imi.bg.ac.rs (A.P.); dianab@imi.bg.ac.rs (D.B.)

**Keywords:** mesenchymal stromal/stem cells, senescence, inflammaging, tumor, microenvironment

## Abstract

As an organism ages, many physiological processes change, including the immune system. This process, called immunosenescence, characterized by abnormal activation and imbalance of innate and adaptive immunity, leads to a state of chronic low-grade systemic inflammation, termed inflammaging. Aging and inflammaging are considered to be the root of many diseases of the elderly, as infections, autoimmune and chronic inflammatory diseases, degenerative diseases, and cancer. The role of mesenchymal stromal/stem cells (MSCs) in the inflammaging process and the age-related diseases is not completely established, although numerous features of aging MSCs, including altered immunomodulatory properties, impeded MSC niche supporting functions, and senescent MSC secretory repertoire are consistent with inflammaging development. Although senescence has its physiological function and can represent a mechanism of tumor prevention, in most cases it eventually transforms into a deleterious (para-)inflammatory process that promotes tumor growth. In this review we are going through current literature, trying to explore the role of senescent MSCs in making and/or sustaining a microenvironment permissive to tumor development and to analyze the therapeutic options that could target this process.

## 1. Introduction

Mesenchymal stem/stromal cells (MSCs) are adult stem cells that reside in perivascular stromal compartment of virtually all tissues, with the key role in maintaining tissue homeostasis. They have self-renewal and multi-lineage differentiation abilities, as well as multifaceted regulatory and trophic functions exhibited through the paracrine activity of a wide repertoire of secreted immunomodulatory, anti-inflammatory, angiogenic, and anti-apoptotic factors [1]. Moreover, MSCs express a diverse repertoire of chemokine receptors that help them home preferentially to sites of injury or inflammation [2]. Due to these unique characteristics, but also owing to easy access, low immunogenicity, and lack of ethical issues, MSCs represent a promising cell-therapeutic tools in regenerative medicine and a growing number of diverse pathologies [3]. However, their use is limited by a decline in their regenerative potential with increasing donor’s age, as well as by the need for their expansion in vitro, due to the limited yield of these cells from primary sources. Both biological aging and prolonged in vitro culture can expose MSCs to various genotoxic stresses that activate DNA damage response (DDR) and can ultimately lead to a state of irreversible growth arrest, known as cellular senescence [4]. This impairment of MSC functions contributes to a progressive decrease in tissue maintenance and repair, and the overall deterioration of health, characteristic for the aging process.

Cellular senescence is not an unequivocally deleterious process. Actually, it is essentially an important mechanism for the prevention of potential cancerous cell proliferation, as well as a mechanism for promotion of tissue repair [5]. Senescence can be viewed as a temporally regulated continuum of inflammatory phenotypes that ranges from reversible and beneficial repair responses to injury, to a prolonged low-grade para-inflammatory state that allows clearance of transformation-prone cells, to overt inflammatory senescence-associated secretory phenotype (SASP) that facilitates tumor cell growth [5,6,7]. SASP composition differs depending on cell type, tissue of origin, and stressor, but the common features of SASP are that it sustains and propagates senescence in an autocrine and paracrine manner and induces organism-wide low-grade chronic inflammatory state, termed inflammaging [8]. SASP factors support the generation of myeloid-derived suppressor cells (MDSC), and other immunosuppressive cells (regulatory T cells, M2 macrophages, tolerant dendritic cells, etc.), leading to immunosenescence, and a perpetual loop of inflammaging and tissue degeneration is established [9].

As MSCs tend to home to sites of inflammation and injury, they are regular residents of tumor tissue. Tumor microenvironment (TME), consisting of miscellaneous cell types, including stromal cells and immune cells, has lately arisen as a pivotal participant in tumor development and progression. Among others, MSCs and tumor-associated macrophages (TAMs) create a draw-back loop in which MSCs, via cell–cell contact and paracrine or extracellular vesicle (EV)-mediated transfer mechanisms, implement immunoregulatory effects on tissue-resident macrophages, polarizing them toward M2-like TAMs [10]. Consequently, M2-TAMs modulate the shift of “naïve” MSCs to tumor-derived MSCs with more potent pro-tumorigenic role. Another type of TME cells that can influence tumor development and progression are the cancer-associated fibroblast cells (CAFs). CAFs are the major component of stroma in desmoplastic cancers, the outcome of which is influenced by the interplay between CAFs, MSCs, and cancer stem cells (CSCs) [11].

The role of MSCs in the inflammaging process and the connection between MSC senescence and cancer development is not completely established, although numerous features of aging MSCs, including altered immunomodulatory properties, impeded MSC niche supporting functions, and senescent MSC secretory repertoire are consistent with inflammaging development [12]. Here, we review the available literature with the aim of exploring the role of senescent MSCs in creating and/or sustaining a tumor-supporting microenvironment, and to analyze emerging therapeutic options that target this process.

## 2. Inflammaging and Tumor Development

Broadly speaking, an inflammatory response is engaged whenever tissue malfunctions are detected. Depending on the nature and the degree of tissue malfunction, the magnitude of the inflammatory response can vary significantly, ranging from localized reaction of tissue-resident cells to mobilization of organism-wide inflammatory potential and recruitment of full inflammatory cellular and molecular armory to the site of injury [13]. In that regard, para-inflammation would be a low-grade inflammatory response at an intermediate state between tissue homeostasis and classic inflammation, serving to restore tissue homeostasis upon persistent tissue stress [14].

Indeed, inflammation is a favorable, protective process as an acute immune response to detrimental conditions. While acute inflammation is a self-limiting immune response, since the production of anti-inflammatory cytokines follows the production of pro-inflammatory cytokines [15], in chronic inflammation a multitude of cell deaths occurs as a result of persistence of initiating factors or a loss/disfunction of repair mechanisms [16]. One of the main characteristics of aging is a reduction in the capacity to tolerate chemical, antigenic, and nutritional triggers, giving rise to tissue disfunction and degeneration. This leads to a state of low-grade chronic inflammation called inflammaging, a central component of the aging process [17,18]. Inflammaging is a fundamental risk factor for both morbidity and mortality in elderly people, since most age-related diseases share an inflammatory pathogenesis. Numerous data indicate a common pattern in seemingly different age-related pathologies, like cancer, type 2 diabetes, and cardiovascular diseases [17,18,19]. Indeed, inflammaging is characterized by increased levels of interleukin (IL)-6, tumor necrosis factor (TNF)-α, and inflammatory markers such as C-reactive protein (CRP), main factors of pro-inflammatory state [17]. In addition, defective function of autophagy in aging leads to the accumulation of non-degraded cellular waste-products in the body (damage-associated molecular patterns, DAMP). This activates the innate immune system, primarily macrophages, to secrete pro-inflammatory cytokines and chemokines, including IL-1, IL-6, and IL-8. Consequently, the NF-κB-mediated signaling cascade is activated in immune cells and the state of low-grade chronic inflammation is instigated [20].

The connection between inflammation and cancer was first hypothesized by Rudolph Virchow in the nineteenth century, suggesting that the origin of cancer was at the sites of chronic inflammation, but researchers devoted any attention to this subject only in the last two decades [21]. It has now been determined that chronic inflammation is associated with all stages of cancer development, including support of cancer initiation, cancer progression, and metastatic dissemination [22,23,24]. In the process of cancer initiation, neoplastic transformation occurs as a disruption of tissue homeostasis in the direction of cancer cell formation. This process includes irreversible DNA alterations that can persist indefinitely in normal tissues until the secondary stimulus such as chemical irritants, hormones, or chronic irritation and inflammation as promoting factors appears [24]. Functionally, many promoters of inflammation influence cell proliferation, inflammatory cell recruitment, and production of reactive oxygen species, leading to DNA damage and DNA repair reduction [22,23].

In addition, it has been shown that stimulation of innate immune signaling through activation of pattern recognition receptors and archetypal inflammatory pathways, such as NF-κB and IRF-3, opens chromatin configuration and promotes a state of transient epigenetic plasticity, rendering the cell permissive to reprogramming and transformation [6]. If limited in time and intensity, this could lead to beneficial tissue regeneration or reparative transdifferentiation. Conversely, if not followed by re-acquisition of the original (or alternative, but beneficial) differentiated cell fate, this stem-like epigenetic state has been suggested to lead to the generation of a cancer cell [6].

Hence, inflammation, aging, and cancer are inextricably linked and connected with a deleterious impact on the wellbeing of the elderly. Thus, successful control of inflammaging should include reduction of chronic inflammation without compromising an acute immune response to pathogens and is essential for healthy aging and longevity.

## 3. Immunomodulatory Functions of MSCs and Their Role in Tumorigenesis

**Figure 1:**
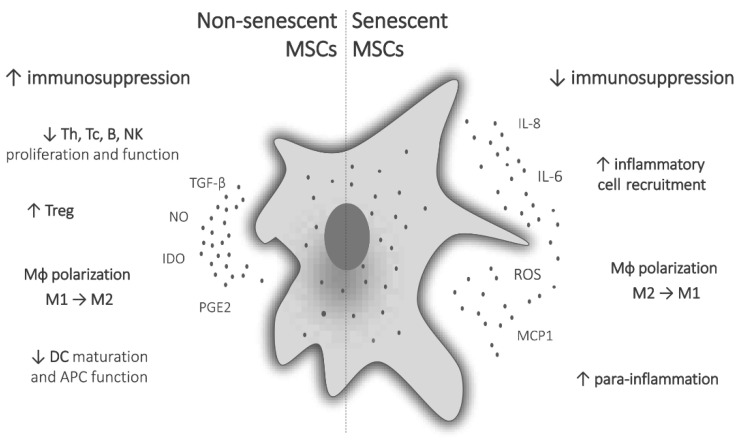
Immunomodulatory functions of non-senescent and senescent MSCs. Immunosuppressive functions of MSCs are expressed through the suppression of proliferation and function of T, B and NK cells, induction of regulatory T cells, polarization of macrophages to anti-inflammatory M2 phenotype, and suppression of DC maturation and antigen-presenting function. Senescent MSCs have reduced immunosuppressive potential, produce pro-inflammatory mediators, recruit inflammatory cells, polarize macrophages to inflammatory M1 phenotype, and lead to systemic low-grade para-inflammation.

The immunomodulatory potential of MSCs is manifested primarily through its influence on functional changes of monocytes/macrophages, dendritic cells (DC), and lymphocytes (Figure 1) [25,26]. It has been demonstrated that MSCs are able to suppress both CD4+ and CD8+ T-cell proliferation, cause a shift in T-helper (Th) profile from Th1 to Th2, induce regulatory T cells (Treg), suppress B-cell functions, and inhibit natural killer (NK) cell proliferation and cytotoxic activity. They also promote the polarization of macrophages toward the anti-inflammatory M2 phenotype, suppress migration and maturation of DC and impede their antigen-presenting potential [27]. It was also shown that MSCs can drive the generation of MDSCs [28,29].

MSCs exert their action through direct cell-to-cell contact or via secretion of soluble bioactive molecules, primarily indoleamine 2,3-dioxygenase (IDO), nitric oxide (NO), transforming growth factor β (TGF-β), IL-10, prostaglandin E2 (PGE2), tumor necrosis factor α-stimulated gene 6 protein (TSG-6), hepatocyte growth factor (HGF), etc. [30,31]. More recently, EVs are increasingly being recognized as important mediators of MSCs’ biological functions. These are small lipid-coated globules containing biologically active proteins, lipids, and nucleic acids (DNA and RNA fragments, micro RNAs) originating from the parental cell, which convey biological properties of the cell, and could be used as cell-free therapeutics, as human MSCs have great potential in the mass production of EVs [32,33].

The nature of the immunomodulatory activity of MSCs is not invariably suppressive and depends essentially on the microenvironment they are exposed to [31,34]. Depending on environmental cues, MSCs can be polarized to pro-inflammatory phenotype, which is, by the analogy with macrophages, termed MSC1, or anti-inflammatory, MSC2 phenotype [35]. For example, in the presence of a Toll-like receptor 4 (TLR4) ligand and low levels of pro-inflammatory cytokines, like IFN-γ, MSCs produce pro-inflammatory cytokines and assume immunostimulatory function [36]. On the other hand, anti-inflammatory phenotype depends on high levels of acute pro-inflammatory signals, and MSCs that are not primed by a sufficient amount of pro-inflammatory factors fail to activate adequate immunosuppressive mechanisms and to suppress proliferation and function of immune cells [37]. In that respect, persistent low amounts of pro-inflammatory factors and comparable levels of anti-inflammatory factors in chronic inflammatory microenvironment drive MSCs toward immunostimulatory phenotype and hinder the resolution of the inflammation [38].

This dual role of MSCs is also evident in tumors. As part of TME, MSCs can play both pro and anti-tumorigenic roles, depending on numerous complex factors, such as tissue of origin, secretome, nature of interactions with cancer and host immune cells, type of cancer cells, and specific in vivo or in vitro condition [39]. Waterman et al. have shown in their in vitro and in vivo models that MSC1 and MSC2 have divergent effects on cancer growth and metastasis. Lipopolysaccharide-primed MSC1 showed a suppressive effect on proliferation, migration, and invasion of tumor cells and attenuated tumor growth and metastasis in mouse tumor models, whereas MSC2, primed with TLR3 agonist poly(I:C), had the opposite, tumor-supporting effects in these model systems [40]. The supportive mechanisms of MSCs on tumor growth are numerous, and apart from the suppression of the immune response against the tumor, include inhibition of tumor cell apoptosis, promotion of cancer stemness and drug resistance, induction and support of angiogenesis, epithelial-to-mesenchymal transition (EMT), and promotion of tumor metastases [41]. MSCs can also differentiate to CAFs that also secrete a plethora of factors to foster cancer stem cells, tumor growth, and invasion [11]. Yet another mechanism of tumor promotion involving MSCs is the process of cell–cell fusion between cancer cells and MSCs leading to the generation of hybrid cells exhibiting cancer stem cell characteristics [42,43]. Conversely, it has also been reported in a number of studies that MSCs can suppress tumor growth by inducing inflammatory cell infiltration, suppression of angiogenesis, induction of cell cycle arrest and apoptosis in tumor and endothelial cells, and inhibition of key signaling pathways involved in tumor cell survival, invasion and migration, like Wnt/β-catenin pathway [41].

Evidently, the role of MSCs in tumor development is not straightforward, and there is still no clear answer to what makes MSCs pro-tumorigenic or anti-tumorigenic. Deeper understanding of factors leading to the promotion of tumor growth or its suppression by MSCs is imperative for their safe and successful application as cell-based therapeutics or for potential manipulation of MSCs with the aim of targeting tumor cells and modulating TME to be unfavorable for tumor development. One of the factors that influences the behavior of MSCs to a great degree is senescence, and in the next sections we will go deeper into the role of this phenomenon in carcinogenesis and tumor progression.

## 4. MSC Senescence

During their prolonged replicative lifetime, MSCs are exposed to numerous endogenous and exogenous stressors that can damage a cell’s genome, like metabolic processes, oxidative stress, physical and chemical agents [44]. As a reaction to these genotoxic stresses, MSCs initiate DNA damage response (DDR) mechanisms to try to repair these damages and, if unsuccessful, to induce differentiation, programmed cell death, or permanent cell cycle arrest, i.e., cellular senescence [44,45]. Human MSCs are relatively resistant to damage-induced apoptosis and preferentially go to cell cycle arrest upon genotoxic injury [46,47]. Telomere shortening, chromatin disorganization, DNA double-strand breaks, and other types of DNA damage, activate DDR proteins, like ataxia telangiectasia mutated (ATM), or tumor suppressors retinoblastoma (Rb) and p53, which activate cyclin-dependent kinases p21 and p16, respectively, ultimately leading to senescence [44,45]. Strong mitogenic signals by oncogenes or overexpressed pro-proliferative genes can also induce cellular senescence [45,48]. In addition to in vivo, replicative senescence of MSCs can be acquired spontaneously in long-term cultures during in vitro expansion that leads to artefactual aging of MSCs. Considering that there is limited direct evidence of senescent MSC characteristics in an aging organism, data gathered from cultured, replicative senescent MSCs, can be fairly extrapolated to aging MSCs in vivo, since differential gene expression in MSC from aged individuals has been shown to correlate to that of in vitro senescent MSCs, indicating the similarity of the aging process in vitro and in vivo [49].

One of the hallmarks of senescence is excessive secretion of a plethora of bioactive molecules, mostly proteins, collectively named senescence-associated secretory phenotype (SASP). This comprises different pro-inflammatory cytokines (IL-6, IFN-γ, TNF-α), chemokines (IL-8, MCP-1, GROα), growth factors (FGFb, HGF, GM-CSF), proteases (MMPs, TIMP-2, uPA), soluble adhesion molecules and receptors (ICAM, VCAM, uPAR, EGFR), extracellular matrix (ECM) components (fibronectin, laminin), and some non-protein small molecules (NO, PGE2, miRNAs) [7,45,50,51]. Sepulveda et al. have identified 27 proteins (of 51 analyzed) that were present in significantly higher amounts in conditioned medium of radiation-induced senescent MSCs compared to the control cells [51]. Peffers et al. did a much broader proteomic analysis and identified 118 (of 777 analyzed) differentially expressed proteins in MSCs from old donors, of which 116 were in higher, and 2 in lower levels than in MSCs from young donors [50]. These proteins are involved in antioxidant regulation, metabolism, transcriptional regulation, cell migration, proliferation, and survival. Although a localized and time-limited SASP can promote tissue regeneration, pronounced and persistent SASP is associated with systemic inflammation, disrupted tissue architecture, and tumor promotion [52].

Senescent cells also produce excessive cellular waste and DAMP, like S100A proteins, heat shock proteins, and advanced glycosylation end products, which activate TLRs and other innate immune cell receptors, inducing and perpetuating chronic inflammatory state, i.e., inflammaging [53].

In addition to the SASP, senescent MSCs exhibit numerous changes in morphology, phenotype, differentiation capacity, migration, and function [54,55]. Senescent cells, including MSCs, reveal enlarged and flattened morphology, exhibit increased levels of ROS, NO, and senescence-associated β-galactosidase (SA-β-gal) activity, foster characteristic nuclear structures, persistent DNA damage foci (PDDF) and senescence-associated heterochromatin foci (SAHF), and display distinctive changes in gene and protein expression [45,49,50,54,56,57,58,59]. A study that compared MSCs from young (2–3 mo) and old (23–24 mo) C57Bl/6 mice has shown that aged MSCs have reduced migration, proliferation, and angiogenic potential compared to MSCs from young animals [55]. Genome profile expression analysis reported over 4000 genes differentially expressed in senescent human MSC compared to control [51], showing a predominant enrichment for biological functions involved in gene expression, cell cycle, and cancer. Examination of human bone marrow (BM)-derived MSC intra- and extra-cellular changes induced by senescence revealed that, due to the establishment of a senescent phenotype, the migratory potential of BM-MSCs is significantly reduced in both 2D and 3D models [60]. Several factors related to the cellular changes during senescence contribute to lower motility. Namely, with the increase of actin stress fibers and decrease of microtubule-binding proteins, intracellular mechanics becomes more homogenous, leading to the establishment of enlarged, non-contractile, static phenotype that provides the viability of senescent cells and the ability to produce SASP factors. Due to cytoskeletal rearrangement, cellular polarization is also altered, contributing to the nuclear deformation and slower migration in both 2D and 3D systems [60].

Differentiation potential of aged and senescent MSCs is altered as well. Although there are some conflicting reports, increasing evidence suggests that the osteogenic capacity of aged MSCs is compromised, while adipogenesis is not so much affected, or is even enhanced [45,54,61,62,63]. On the other hand, it was shown that aging impairs beige adipocyte differentiation of MSCs in adipose tissue and that it was linked to the reduction in Sirtuin 1 (Sirt1) level in aged adipose tissue-derived MSC [64]. It is also important to note that disfunction of adipose tissue itself during aging is responsible for metabolic alterations and multiorgan damage associated with inflammaging, and that white adipose tissue is a major source of cytokines and other pro-inflammatory mediators [65,66,67].

Aging and senescence also influence the immunomodulatory properties of MSCs (Figure 1). Namely, the suppressive activity of BM and umbilical cord (UC)-derived MSCs on mitogen-stimulated T-cell proliferation was found diminished upon long-term cultivation [68,69]. Moreover, the MSCs’ inhibitory effect on the secretion of proinflammatory cytokines (IFN-γ and TNF-α), as well as anti-inflammatory IL-10 by T cells was decreased [68]. On the other hand, senescent MSCs expressed increased levels of proinflammatory mediators, including IL-6 and IL-8 [51], while the stimulatory effect of IFN-γ and TNF-α on the production of anti-inflammatory prostaglandin E2 (PGE2) and its initiatory enzyme cyclooxygenase-2 was reduced in late-passage MSCs [70]. Similarly, MSCs derived from BM and adipose tissue of old animals have been shown to exert a weaker capacity to suppress the proliferation of activated T cells [71], while MSCs from periodontal ligament displayed reduced immunosuppressive effect in older individuals [72]. Aside from affecting lymphocytes, MSC senescence has an impact on the functions of macrophages and MDSC. Macrophages are key players in the process of inflammaging, and many SASP factors influence their recruitment, activation, and polarization [73]. Aging MSCs alter their impact on macrophage polarization. A macrophage cell line, RAW264.7, upon co-cultivation with aged MSCs, increased mRNA expression of TNF-α, characteristic of M1 phenotype, contrary to macrophages cultured with young MSCs, which increased the expression of M2-related genes, arginase-1 and IL-10 [74]. Inflammatory mediators of SASP may also trigger the generation of MDSC, cooperatively enhance the immunosuppressive network, leading to immunosenescence and inflammaging [29,75].

A limited number of in vivo studies reported abrogated protective effect of senescent MSCs on inflammatory diseases in experimental mouse models (mouse experimental colitis and LPS-induced lethal endotoxemia) [51,70]. Moreover, Sepulveda et al. identified four genes that are differentially regulated in radiation-induced in vitro senescent MSCs, as well as in MSCs that failed to produce a therapeutic effect in a GVHD clinical trial in vivo, when compared to control, non-senescent, and therapeutically effective MSCs. In addition, a recent study by Lee et al. showed that inflammation-induced senescence in synovial fluid MSCs of rheumatoid arthritis (RA) patients attenuated their immunomodulatory properties and protective anti-arthritic capacity in an RA animal model [76]. 

The niche-supporting function of MSCs is highly important for the maintenance of BM microenvironment and effective hematopoiesis. Recent literature data indicate that impaired hematopoiesis in an aged organism has been promoted in large part by increased inflammation and induction of inflammaging process in BM hematopoietic niche. Increased secretion of IL-6 and TGF-β by aging BM stroma contributes to age-dependent changes to the composition and function of the hematopoietic stem cell compartment [77,78]. Higher levels of SASP factors in aged BM-MSCs, including, IL-1, IL-6, IL-8, and MCP-1, have been found to impair hematopoietic stem cells clonogenicity [79]. Therefore, abnormal crosstalk between HSCs and MSCs caused by alterations of aged MSCs has been proposed as a possible cause of premalignant processes like myelodysplastic syndrome in aged individuals [80,81,82]. Furthermore, SASP-driven mutations in genes related to dysregulated myelopoiesis, such as additional sex comb-like 1 gene (ASXL1) or ten-eleven translocation 2 gene (TET2), in BM-MSCs may lead to age-associated disturbances in hematopoietic niche-supportive function and pathologic myelopoiesis [80,83,84]. The disturbed hematopoietic microenvironment and skewed hematopoiesis toward myeloid lineages in the elderly could also be attributed to aged MCS-biased adipogenesis, since adipocytes have been shown to be negative regulators of hematopoiesis, especially lymphopoiesis [85,86].

To summarize, cell senescence has deep impact on MSCs phenotype and function, from its cytoskeletal structure and secretory profile to its differentiation capacity, immunomodulatory potential, and niche-supporting functions. But how these changes influence TME and tumor development? We will analyze that in the next section.

## 5. Role of MSC SASP in Forming Tumor Microenvironment

**Figure 2:**
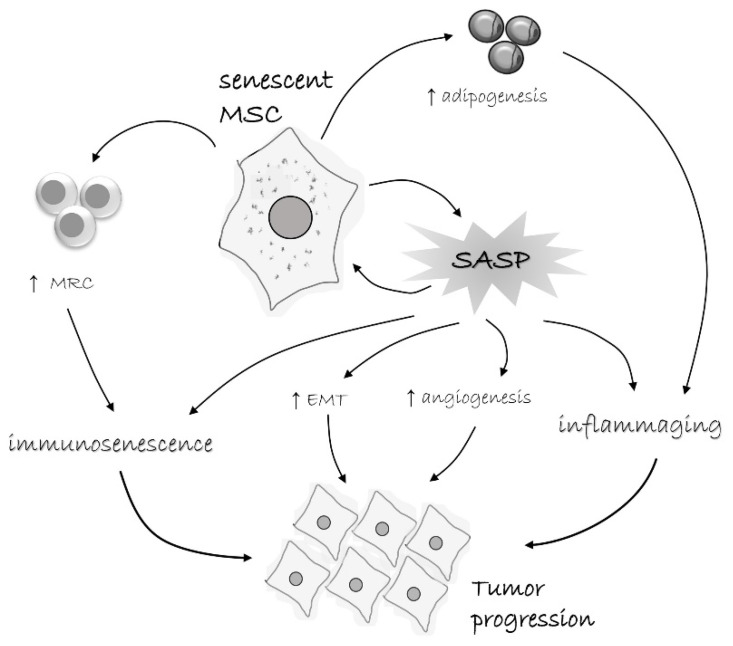
Tumor-promoting characteristics of senescent MSCs. Senescent MSCs acquire senescence-associated secretory phenotype (SASP) and secrete a range of pro-inflammatory cytokines, chemokines, growth factors, pro-angiogenic, anti-apoptotic, and other bioactive molecules. Senescent MSCs also have biased differentiation capacity toward adipocytes, which by themselves produce diverse pro-inflammatory factors. All this lead to a low-grade para-inflammatory state. On the other hand, these factors induce immunosenescence, but also lead to the induction of myeloid regulatory cells (MRC), like MDSC, M2 macrophages, and tolerogenic DC. This creates a tumor-permissive environment and together with other pro-tumorigenic effects, like stimulation of adipogenesis and epithelial-to-mesenchymal transition (EMT), supports tumor progression.

The tumor microenvironment comprises various cell types, including stromal and immune cells, and non-cellular components, such as ECM, EVs, and soluble factors. Through complex interactions with cancer cells, TME significantly influences the process of tumor initiation, development, and progression [87,88,89]. Although it is known that, as a component of TME, MSCs also contribute to tumor growth [90,91], the influence of senescent MSCs on cancer progression has not yet been elucidated (Figure 2). Therefore, in this section, we aim to review and discuss literature data on how specific features of senescent MSCs can contribute to the tumor progression. 

As the SASP acquisition is the general hallmark of senescent MSCs, studies mostly investigated the effects of MSC secretome on cancer cells through examination of the MSCs’ conditioned medium (CM) effects. However, results are variable and dependent on MSC/cancer cell source, senescence type induction, or culture conditions showing both pro- and anti-tumorigenic influence. Indeed, the molecular spectrum of SASP is very broad, diversified, and context-dependent, but some proteins are almost invariably reported as constituents of senescent cells’ secretome [7]. The main culprit among SASP factors associated with the tumor-promoting activity of senescent MSCs is IL-6. It has been shown by numerous studies that this pleiotropic cytokine, derived from senescent stroma, stimulates proliferation, migration, and chemoresistance of tumor cells [58,75,92]. Namely, CM of senescent UC-MSCs was shown to possess tumor-promoting features stimulating proliferation of breast cancer cell lines MCF-7 and MDA-MB-231, as well as their migratory potential in the in vitro trans-well co-culture system. As a potential mechanism involvement of increased IL-6 secretion by senescent MSCs along with STAT3 activation in cancer cells has been proposed. In addition to the in vitro experiments, injection of MDA-MB-321 cancer cells along with senescent MSCs in the xenograft model showed stimulated formation of solid, palpable tumor nodules (initiation), as well as increase in tumor size and weight (i.e., tumor growth) and vascularization, confirming pro-tumorigenic effects of senescent MSCs on breast cancer [92].

In addition, Li et al. examined the effects of CM of adipose tissue-derived MSCs (AT-MSCs) on human colorectal cancer cells, LoVo. They have compared the effects of senescent AT-MSCs from late passages (P30) to those from early passages (P3) and demonstrated an increased stimulatory effect of CM from senescent AT-MSCs on the proliferation of colon cancer cells that were dependent on galectin-3 production [93]. Galectin-3 is also shown to promote tumorigenesis in various solid tumors and hematologic malignancies and increased galectin-3 levels are reported in the bone marrow of aging mice [94,95].

In contrast to these studies showing tumor-stimulative effects of senescent MSCs, the secretome of senescent AT-MSCs promoted senescence or apoptosis of ARH-77 multiple myeloma cells [96]. Moreover, the secretome of senescent AT-MSCs induced reduction of Ki67 positive ARH-77 cells confirming their anti-tumorigenic effects. Further exploration of senescent BM- and AT-MSCs secretome, induced by H_2_O_2_, doxorubicin treatment, X-ray irradiation, and replication, revealed the activity of three key signaling pathways including MMP2–TIMP2, IGFBP3–PAI-1, and peroxiredoxin 6–ERP46–PARK7–cathepsin D–major vault protein, that may be involved in the paracrine interactions with adjacent cells [97]. Interestingly, the same authors showed that the effects of senescent AT-MSCs secretome also depend on the MSCs’ interactions with cancer cells. Namely, they reported that senescent AT-MSCs, after priming with myeloma cells, lose the ability to reduce the number of Ki-67 positive ARH-77 cells, indicating that changes in the secretome composition prevent the anti-cancer abilities of MSCs [96]. 

Conflicting data reported on MSCs’ SASP effects on tumor growth can be explained by the cancer cell property to re-educate senescent MSCs to secrete factors that support their survival and obviate anti-cancer effects of SASPs possibly through downregulation of pathways associated with senescence, apoptosis, and metabolic processes, and upregulation of ECM signaling involved in cancer growth and metastasis. Moreover, a recent study by Alessio and co-workers indicated that the cancer stage can also influence MSCs’ secretome potential to inhibit cancer cell proliferation and stimulate their cell cycle arrest that leads to senescence [98]. Namely, they obtained evidence that SASPs of senescent MSCs induced by H_2_O_2_ or low X-ray doses (acute senescence) stimulated the senescence of immortalized PTN2 prostate cells, reducing their proliferation and number of cells in the S-phase, while not affecting the PC3 metastatic prostate cancer cells. These data suggest that SASP derived from acute senescent cells might target pre-tumorigenesis events.

In addition to the influence of MSC secreted factors, as an integral part of the tumor microenvironment, recent research indicates the importance of considering the senescent MSC effects on ECM remodeling in cancer progression [60]. Namely, although senescent MSCs change their morphology and acquire reduced motility, they continue to produce ECM components, MMP inhibitors (TIMPs), and crosslinking agents (transglutaminase and LOX), resulting in the altered ECM structure that affects cancer cell behavior. Findings of the quantitative analysis of single-cell migration from spheroids into the surrounding ECM in a 3D matrix model of MSCs and breast cancer cells (BCCs) showed that altered ECM production by senescent MSCs is the mechanism that influences the behavior of BCCs leading to their increased proliferation and motility. Moreover, matrix remodeling and secretion of soluble factors by senescent MSCs may also have an influence on pre-senescent MSCs, enhancing their motility and consequently tumor invasiveness. Overall, the data collected in this study suggest that senescent MSCs can support the progression of breast cancer by ECM remodeling.

On the other hand, tumor cells and tumor microenvironment, as well as therapeutic interventions can induce cell senescence in surrounding MSCs. Ridge et al. have observed that secretomes from prostate cancer cell lines induced an altered phenotype of cocultured MSCs, which resulted in increased secretion of pro-inflammatory cytokines (IL-6, IL-8, MCP-1, IL-11, osteopontin, MIF, FGF-2), and reduced proliferation and migration capacities, as well as an increase in SA-β-gal positive MSCs in the coculture [99]. Moreover, when human BM-MSCs from multiple myeloma patients were analyzed by André et al., a senescent profile with profound alterations in their characteristics was demonstrated, including increased cell size, reduced proliferation capacity, decreased osteogenic potential, impaired immunosuppressive capacity, and a distinct gene and protein expression profile [100]. Out of 30 examined proteins, they found increased secretion of IL-6, IL-8, MCP-1, MIP-1a, MIP-1b, MMP-2, MMP-9, OPG, RANTES, TIMP-1, TIMP-2, VEGF, BDNF, HGF, and IGF-II in the patients’ BM-MSC. As already mentioned, chemotherapeutics and radiation can induce persistent DNA damage and consequent senescence in MSCs [46,47]. These therapy-associated genomic alterations and induction of SASP in MSCs could be one of the factors that make a favorable microenvironment for the persistence of the tumor or development of secondary malignancies [101,102].

All these data clearly indicate that senescent MSCs are important agents in the formation of a tumor-permissive microenvironment and that tumor cells and TME itself also induce senescent phenotype in MSCs and other tumor-infiltrating cells. Hence, targeting senescence of MSCs and/or manipulating it in a way to work against the tumor could be a promising strategy in cancer therapy.

## 6. Therapeutic Implications of MSC Senescence Targeting

To ensure effective therapy with MSCs, management of cell aging needs to be taken into account. Based on previous knowledge and discoveries on mechanisms of senescence, different strategies for the rejuvenation of MSCs are being developed. According to the available literature, so far pharmacological, genetic, and cytokine approaches have been investigated (Table 1). 

Senolytic drugs, transiently disable SCAPs (senescence associated anti-apoptotic pathways) causing apoptosis of senescent cells [103]. Their therapeutic effects on different diseases have been investigated in many clinical trials and early results of some suggest improvement of the state of patients. A couple of senolytics were investigated as candidates for the treatment of senescent MSCs. Namely, a flavonoid quercetin increased the proliferation of old rat BM-MSCs, upregulated the osteogenesis, and helped with the elimination of senescent cells in vitro, although via an unknown mechanism [104]. When added in a combination with dasatinib, a tyrosine kinase inhibitor, it improved the osteogenic capacity of aged mouse BM-MSC both in vitro and in vivo [105]. Dasatinib itself, on the other hand, decreased senescence in AT-MSC of patients with preeclampsia and resulted in improved MSC angiogenic potential, though authors indicate the low number of samples as a limitation of the study [106]. As for navitoclax, a Bcl-2 family protein inhibitor (also known as ABT-263), a reducing effect on senescence cell burden of mouse BM-MSC was found, but in parallel with a decreased osteogenic potential of these cells, both in vitro and in vivo [107]. The authors concluded that the use of this drug for the treatment of age-related musculoskeletal dysfunction and bone loss could be potentially harmful. Its potency was also questioned in the work of Grezella et al. where ABT-263 significantly reduced the proportion of senescent human BM-MSCs, but not rejuvenating them in terms of telomere length or epigenetic senescence signature [108]. Therefore, having in mind the limitations of senolytics’ potential use, their application has to be further investigated.

**Table 1:** Different strategies for the treatment of senescence in MSCs.

Th Agent/Approach	MSC Type	Rejuvenating Effect	Reference
**Senolytics**			
Quercetin	rat BM-MSCs	Elimination of senescent cells in vitro; ↑ proliferation, ↑ osteogenesis, ↓ adipogenesis; no change in migration	[104]
Quercetin + dasatinib	mouse BM-MSC	↓ Number of senescent cells; ↑ proliferation, improving the osteogenic capacity of old BM-MSC in vitro and in vivo	[105]
Dasatinib	human AT- MSC	↓ Senescence in MSCs of patients with preeclampsia; improved MSC angiogenic potential	[106]
Navitoclax (ABT-263)	mouse BM-MSC	↓ Senescence cell burden, ↓ in vitro and in vivo osteogenic potential of MSCs	[107]
	human BM-MSCs	Mild ↓ proportion of senescent cells; no effect on telomere length or epigenetic senescence signature	[108]
**Genetic manipulation**			
NDNF overexpression	human BM-MSCs	↓ Cell senescence and apoptosis ↑ proliferation and angiogenesis in old cells; improved cardiac function in mice after myocardial infarction	[109]
	human AT-MSCs	Improving proliferation and migration capacity; preserved cardiac function and reduced scar formation in vivo	[110]
Apelin overexpression	human AT-MSCs	↑ Autophagy; enhanced paracrine effects; promoted cardioprotection following myocardial infarction in mice	[111]
MIF overexpression	human BM-MSCs	Inhibiting cellular senescence of aged cells; activating autophagy improving their survival under serum deprivation/hypoxia in vitro; enhanced cardioprotection after myocardial infarction	[112]
Silencing of miR-195	mouse BM-MSCs	Reduced number of senescent cells in vitro, induced telomere re-lengthening, restored proliferation and expression of anti-aging factors Tert and Sirt1; reduced infarction size and improved left ventricular function	[113]
Inhibition of miR-199a-5p	IPF-MSCs	Promoted autophagy and ameliorated senescence; transplantation of anti-miR-199a-5p-IPF-MSCs increased their capacity to prevent induced lung fibrosis progression in mice	[114]
Inhibition of mir-188	human and mouse BM-MSCs	Reduced age-associated processes, injection of antagomiR-188 into bone marrow stimulated bone formation and decreased bone marrow fat in aged mice	[115]
Downregulating of lnc-CYP7A1-1	human BM-MSCs	↓ Cell senescence, ↑ proliferation, ↑ migration, ↑ survival; in vivo improved cardiac function in mice after MI	[116]
Silencing lncRNA-p21	mouse BM-MSCs	Enhanced cell growth and paracrine function, ↓ oxidative stress	[117]
5-AZA	human AT-MSCs	↑ Proliferation ↓ ROS accumulation, ↓ DNA methylation status ↑ BCL-2/BAX ratio	[118]
	human AT-MSCs	Improving proliferation and osteogenic differentiation potential; Induced Expression of TET2 and TET3, ↑ Nuclear 5 hmC Levels	[119]
Tetramethylpyrazine	mouse BM-MSCs	Inhibited senescence via histone-lysine N-methyltransferase enzyme EZH2; creating an anti-inflammatory and angiogenic environment in the bone marrow of aging mice	[120]
**Cytokine treatment**			
MIF	rat BM-MSCs	Pretreatment enhanced growth, paracrine function and survival.; inhibited hypoxia/serum deprivation-induced apoptosis	[121]
	rat BM-MSCs	Pretreatment improved the proliferation rate, viability, paracrine function, telomere length, and telomerase activity of DOXO-treated MSCs; ↓ oxidative stress, ↑ activity of SOD.	[122]
**Extracellular vesicles (EVs)**			
Infant AT-MSC-derived EVs	human AT-MSCs	Promoting proliferation, ↓ number of β-gal-positive cells, inhibiting the elevation of ROS by upregulating the expression of SOD1 and SOD3; induced the ability of elderly MSCs to decrease necrotic area in type 1 and type 2 diabetic mice	[123]
iPSC-derived EVs	human BM-MSCs	Reduced oxidative stress	[124]
UC-MSCs-derived EVs	human BM-MSCs	Enhanced proliferation, mobility, and paracrine activity; better cardiac function, more neovascularization, and less scar formation after transplantation into infarct heart	[125]
Mouse ESC-derived EVs	human PLAC-MSC	Enhanced proliferation and stemness; ↓ SA-β-gal activity; ↓ DNA damage foci, ↓ p16 and p53 mRNA expression; enhanced the retention of MSCs in the mouse cutaneous wound sites and facilitated the cutaneous wound healing process	[126]

Abbreviations: BM-MSC: Bone marrow mesenchymal stem cells; AT-MSC: Adipose tissue mesenchymal stem cells; IPF-MSCs: adipose derived MSC from patients with idiopathic pulmonary fibrosis; PLAC-MSC: placental mesenchymal stem cells; NDNF: neuron-derived neurotrophic factor; MIF: macrophage migration inhibitory factor; 5-AZA: 5-azacytidine; MI: myocardial infarction; ROS: reactive oxygen species; DOXO: doxorubicin; SOD: superoxide dismutase; iPSC: induced pluripotent stem cells; ESC: embryonic stem cells.

Recent scientific data suggest that genetic manipulation of senescent MSCs was shown to be a more promising method, since many available tools enable researchers to identify molecules involved in senescence regulation and to successfully utilize them. For instance, neuron-derived neurotrophic factor (NDNF) was recognized as one such molecule, and its overexpression in old human MSCs could rejuvenate these cells in vitro by, e.g., decreasing cell senescence and apoptosis in BM-MSCs [109] and improving proliferation and migration capacity of AT-MSCs [110]. Moreover, the engraftment of NDNF-overexpressing old MSCs was shown to improve cardiac function in mice after myocardial infarction [109,110]. Similarly, overexpression of apelin, an endogenous ligand that might be involved in the regulation of MSC senescence, rejuvenated human AT-MSCs by increasing autophagy and promoted cardioprotection following myocardial infarction in mice [111]. Increased expression of macrophage migration inhibitory factor (MIF) also rejuvenated aged human BM-MSCs by activating autophagy and improving their survival under serum deprivation/hypoxia in vitro [112]. Furthermore, transplantation of MIF-aged MSCs in ischemic hearts of rats enhanced cardioprotection after myocardial infarction. 

Many non-coding RNA molecules were shown to regulate senescence in MSCs and thus attracted attention as potential targets. Namely, inhibition of miR-195, a small non-coding regulatory RNA molecule, reduced the number of mouse senescent BM-MSCs in vitro, induced telomere re-lengthening, and restored their proliferation and expression of anti-aging factors Tert and Sirt1 [113]. Moreover, transplantation of old MSCs with knocked-out miR-195 reduced infarction size and improved left ventricular function. Another study showed that miR-199a-5p, found in the serum of idiopathic pulmonary fibrosis patients, induced senescence of human AT-MSC but its inhibition promoted autophagy and ameliorated senescence via the Sirt1/AMPK signaling pathway [114]. As expected, transplantation of anti-miR-199a-5p-IPF-MSCs increased the ability to prevent the progression of pulmonary fibrosis in bleomycin-treated mice. Chang-Jun Li et al. demonstrated mir-188 involvement in differentiation shift of aged BM-MSCs into adipocytes rather than osteoblasts, which leads to progressive accumulation of fat and bone loss [115]. Significantly, in mir-188 knock-out mice, these age-associated processes were reduced, and injection of antagomiR-188 into bone marrow stimulated bone formation and decreased bone marrow fat in aged mice. 

Moreover, Dong et al. discovered that long non-coding RNA (lnc), lnc-CYP7A1-1, stimulated senescence in human BM-MSCs, and its downregulation subsequently improved regenerative capacities and decreased cell senescence in vitro [116]. Consequently, transplantation of old human BM-MSCs with downregulated lnc-CYP7A1-1 improved cardiac function in mice after myocardial infarction. Similarly, silencing of lncRNA-p21 in aged mouse BM-MSCs enhanced cell growth and paracrine function, and decreased oxidative stress probably through the Wnt/β-catenin signaling pathway [117]. An interesting aspect of targeting senescence was explored also at the epigenetic level. For instance, DNA methyltransferase inhibitor 5-azacytidine (5-AZA) was able to rejuvenate human AT-MSCs by reducing reactive ROS accumulation, increasing BCL-2/BAX ratio [118], and improving osteogenic differentiation potential [119]. An alkaloid tetramethylpyrazine on the other hand inhibited senescence in mouse BM-MSCs by regulating a histone-lysine N-methyltransferase enzyme EZH2 [120].

Biologically active molecules such as cytokines were shown to have rejuvenating roles in MSC aging, and this approach might be more translatable to humans. The aforementioned MIF is a proinflammatory cytokine suggested as a good candidate for rejuvenation of MSCs given that it modulates age-related processes in these cells. Pre-treatment of aged rat BM-MSCs with MIF enhanced their growth, paracrine function, and survival, possibly through increased CD74-dependent phosphorylation of AMPK and FOXO3a [121]. The authors confirmed the anti-senescent function of MIF in a later study where it improved telomere length and telomerase activity, inhibited oxidative stress, and activated the PI3K/Akt signaling pathway in doxorubicin-induced senescent rat BM-MSCs, suggesting this could be a good therapeutic strategy for cancer patients under doxorubicin treatment [122].

Extracellular vesicles are suggested as a possible effective cell-free approach for treating aging and degenerative diseases [127]. As already mentioned, these vesicles carry proteins, lipids, DNA, RNA, and other components and are important intercellular mediators that regulate different cellular processes [32,33]. It was demonstrated that human AT-MSCs from aged donors were rejuvenated by infant AT-MSC-derived EVs by promoting proliferation, reducing the number of β-gal-positive cells, and inhibiting the elevation of ROS in vitro, as well as by inducing their ability to decrease the necrotic area in both type 1 and type 2 diabetic mice [123]. Similarly, purified EVs from induced pluripotent stem cells alleviated aging phenotypes of both replicative and induced senescent human BM-MSCs [124]. Authors noticed these EVs contained a high concentration of intracellular antioxidant proteins peroxiredoxins and subsequently reduced oxidative stress in the induced senescent cells. Ning Zhang et al. discovered that EVs from UC-MSCs also reduced senescence phenotypes and improved activities of aged human BM-MSCs and enhanced their function for myocardial repair by transferring exosomal miR-136 and downregulating apoptotic protease activating factor 1 (Apaf1) [125]. In addition, EVs that were derived from mice embryonic stem cells showed the capability to rejuvenate senescent human placental MSCs via IGF1/PI3K/AKT pathway and enhanced their therapeutic effects in vivo [126].

Some new prospective approaches are emerging in recent years, such as combination of senolytic drugs with classical chemotherapeutics or cancer immunotherapies to maximize the effect of conventional therapies and to decrease cancer resistance, or modifying the SASP composition by using “senomorphics” to control or prevent the chronic inflammation and its detrimental consequences, while preserving or boosting the anti-tumor immune response [128,129]. Targeting SASP components, such as the EGFR ligand amphiregulin, implicated in senescence-associated immunosuppressive TME in numerous tumors, showed promising results in minimizing chemoresistance and restoring immunocompetence in a mouse model of prostatic cancer treated with genotoxic therapeutics [130].

However, in the context of cancer therapy, there is still a knowledge gap regarding how to achieve effective implementation of senescence-blocking approaches targeting MSCs within TME. Namely, further perplexity arises from the implementation of pro-senescence therapy based on the exactly opposite process, e.g., selective senescence induction in cancer cells that can limit cancer development [131]. Therefore, as a potentially even more effective strategy, the ‘’two-punch’’ approach has been proposed, where combined utilization of pro-senescence and senolytic therapies are supposed to favor senescence induction in cancer cells along with antitumor immunity, while promoting the removal of the senescent tumor and stromal cells as well. However, considering the complexity of mutual interactions between different stromal and immune constituents of TME with cancer cells, further extensive in vitro and in vivo studies are needed to emphasize the effectiveness and safety of targeting senescent MSCs in cancer treatment.

## 7. Conclusions

Tumorigenesis is a very intricate process in which healthy tissue cells and immune cells engage in interaction with transformed cells in mutual struggle for survival. The impact of senescence and SASP on tumor development is especially complex, ranging from anti-tumorigenic to pro-tumorigenic. The role of MSCs’ senescence in this process is even more obscure. Even the role of non-senescent MSCs in tumor development is not fully elucidated, since it is not known what makes some MSCs tumor suppressors while others tumor promoters. Much of the problem lies in the difficulty of analyzing in situ effects of senescent MSCs, since their relatively low number in tissues imposes a need for in vitro expansion, which itself leads to their artifactual senescence. Even though there are many studies on MSC senescence in the context of tumor development and therapy, which we tried to review in this paper, much more research is needed in order to exploit MSCs’ physiologic and pathophysiologic role in fighting the tumor. The future of anti-cancer treatments should undoubtedly implement strategies to diminish or modify the detrimental effect of MSC senescence on tumor growth and drug resistance.
